# Sample Preparation and Analytical Techniques in the Determination of Trace Elements in Food: A Review

**DOI:** 10.3390/foods12040895

**Published:** 2023-02-20

**Authors:** Leina El Hosry, Nicolas Sok, Rosalie Richa, Layal Al Mashtoub, Philippe Cayot, Elias Bou-Maroun

**Affiliations:** 1 Department of Nursing and Health Sciences, Notre Dame University-Louaize, Zouk Mosbeh, Lebanon; 2 PAM UMR 02 102, Institut Agro, Univ. Bourgogne Franche-Comté, 1 Esplanade Erasme, 21000 Dijon, France

**Keywords:** heavy metals (HMs), analytical techniques, ashing, digestion, toxic elements, atomic absorption spectroscopy (AAS), inductively coupled plasma atomic emission spectroscopy (ICP-AES), inductively coupled mass spectrometry (ICP-MS), X-ray spectrometry

## Abstract

Every human being needs around 20 essential elements to maintain proper physiological processes. However, trace elements are classified as beneficial, essential, or toxic for living organisms. Some trace elements are considered essential elements for the human body in adequate quantities (dietary reference intakes, DRIs), while others have undetermined biological functions and are considered undesirable substances or contaminants. Pollution with trace elements is becoming a great concern since they can affect biological functions or accumulate in organs, causing adverse effects and illnesses such as cancer. These pollutants are being discarded in our soils, waters, and the food supply chain due to several anthropogenic factors. This review mainly aims to provide a clear overview of the commonly used methods and techniques in the trace element analysis of food from sample preparations, namely, ashing techniques, separation/extraction methods, and analytical techniques. Ashing is the first step in trace element analysis. Dry ashing or wet digestion using strong acids at high pressure in closed vessels are used to eliminate the organic matter. Separation and pre-concentration of elements is usually needed before proceeding with the analytical techniques to eliminate the interferences and ameliorate the detection limits.

## 1. Introduction

While there is ongoing discussion about what should be classified as essential, beneficial, or toxic to living beings, and particularly to humans, about 20 of the known elements are qualified today as essential [1]. Trace elements (TEs) are classified as major minerals (macro-minerals) and trace minerals (micro-minerals). These elements have in most cases key biological roles in living organisms. Although the needed quantities of trace elements in the body do not directly indicate their importance or significance, micro-minerals are such that small quantities of these components are required for the organism (compared with major minerals). Dietary reference intakes (DRIs) of minerals give different recommendations, such as EAR (estimated average requirement) and AI (adequate intake), but for vitamins and minerals, two other parameters are preferred: RDA and UL. First, the RDA value (recommended daily allowance) indicates the daily intake of a mineral that satisfies the need of 98% of the population (the minimum of daily intake). Secondly, the UL value (tolerable upper intake level) constitutes the upper limit of daily intake not to be exceeded (the maximum above the risk of adverse effects increases) [2]. UL is defined using two types of experimental data, NOAEL (no-observed adverse effect level) and LOAEL (lowest observed adverse effect level). The UL value is above that of the NOAEL in order to obtain a safety margin. Major minerals include several elements such as calcium (Ca), sodium (Na), magnesium (Mg), phosphorus (P), potassium (K), sulfur (S), and chloride (Cl); while trace minerals include iodine (I), selenium (Se), zinc (Zn), copper (Cu), iron (Fe), molybdenum (Mo), manganese (Mn), cobalt (Co), boron (B), fluoride (F), and chromium (Cr) [3].

To illustrate what defines a trace element (TE) that can be considered a pollutant, let us compare values from the European dietary reference values (DRVs) with PRI (population reference intake or RDA) and UL [4]. For calcium, the PRI-RDA value is 950 mg per day for adults, and the UL for calcium is 2.5 g per day [5]. For the chromium ion (Cr III), an RDA or PRI do not exist but an AI value (adequate intake), a mean usual intake, gives an observed interval intake of 57 to 84 µg/day in adult population. The UL is not defined but a tolerable daily intake (TDI) for Cr(III) has been given as 300 µg/kg of body [6], nearly corresponding to a mean UL value of 21 mg per day. Chromium ion is a useful micronutrient but can also become a pollutant at high concentration. Some other TEs do not appear in the list of 13 mineral nutrients [7], such as lead or mercury. These TEs are addressed by European regulations such as the directive 2010/75/EU on industrial emissions, the directive 2008/50/EC on ambient air quality and cleaner air for Europe, and the directive 2004/107/EC relating to TEs and polycyclic aromatic hydrocarbons in ambient air. Limits of toxicity for the organism or for the environment exist. For lead, the level of poisoning is defined at 250 µg/L of blood for adults according to WHO guidelines [8]. A blood mercury level higher than 5.8 µg/L constitutes an adverse health risk to babies. The Food and Agriculture Organization (FAO) and the World Health Organization have determined a WHO dietary limit level of methylmercury (MeHg) in food: 0.5 mg MeHg/kg for fish and 1 mg MeHg/kg for predatory fish (see Codex Alimentarius of WHO, CODEX 1995) [9].

On the other hand, worldwide pollution by TEs through air, water, and earth poses serious risks to the environment and to the health of human beings [10]. The major sources of TEs are from milling, industrialization, mining, combustion of fossil fuels, and agrochemicals that discharge several types of TEs such as As, mercury (Hg), Chromium (Cr), Cadmium (Cd), nickel (Ni), copper (Cu), cobalt (Co), lead (Pb), and zinc (Zn) into the waters and agricultural soils [10,11]. In addition, TEs contamination of soils may occur due to sewage water irrigation [12], pesticides and fertilizers usage, heavy agricultural equipment operations, erosion, landfill leaching sites, dry and humid deposits of industrial pollutants, incorporation of municipal solid-waste compost, and volcanic activities [13,14]. Fly ash also includes a variety of TEs such as Pb, Zn, Cd, As, Ni, Cu, and Cr, etc. [15].

Knowing that the main cause of human exposure is oral ingestion, the accumulation of TEs may occur from the consumption of products of animal origin (muscle, liver, kidney, milk, egg) and of fish tissues [16,17]. In addition, cultivated soils are the main source of TEs contamination in vegetables used for human consumption. Therefore, because of food safety concerns, the testing of TEs has become a matter of utmost importance and there is an increasing concern about the potential health risks posed by the occurrence of multiple micro-pollutants and TEs in food [16].

There is a need to control TE content in food for nutritional and functional purposes where TE play a physiological role [18]. For example, Cu, Zn, and Mn are naturally present in some enzymes, and iodine (I) is important to produce thyroid hormones. On the other hand, another important reason for monitoring trace elements is food safety. An important number of TE are toxic and their quantities in food are limited and defined by the legislation [19].

Analytical methods must be accurate and sensitive enough for the determination of some TEs in the ppb (µg/kg) level. In this review, we will focus on the analysis of TEs in food. After providing a detailed overview on the main toxic TEs found in food, we will dive more into the analysis strategy that is usually followed for testing these elements. In fact, various methods and techniques are commonly used in trace element analysis, from sample preparation, ashing techniques, and separation/extraction methods to the analytical techniques used. We will also discuss the choice criteria for the different methods/techniques used, the purpose of each method, as well as their corresponding advantages and disadvantages.

## 2. Main Toxic Elements in Food

Dangerous TEs can cause several adverse effects on living beings when they exceed the safe limits. However, some, such as Fe, Co, Mn, Mo, and Zn, are necessary in small quantities to maintain certain physiological processes in living beings [10,20]. The Earth’s crust is one of the major TE resources. It naturally contains a variety of metals and metalliferous Tes that can move to the surface with geological activities, human activities (metallurgy and mining), sewage, wastewater, industrial activities, fuel consumption, fertilizers, and agriculture; this can increase the level of these TEs in soil, air, and water. This contamination with toxic TE pollutants has a wide effect on the food supply chain both directly and indirectly because it affects aquatic life, animals, humans, and plants. It should be noted that contamination with TEs can bioaccumulate over time in living organisms, and can therefore reach toxic concentrations which ultimately cause serious adverse effects [21]. The intake of TE pollutants from contaminated food may lead to several acute and chronic health effects such as damage to the kidneys and lungs, gastrointestinal irritation, fatigue, headaches, nervous system disorders, skin manifestations, cardiovascular damage, muscular pain, and cancer [10,22,23]. 

The dietary exposure to TEs via food consumption is usually evaluated by using the estimated daily intake (EDI), which considers the average level of these TEs along with the respective rate of consumption for adults. Then, the calculation of the estimated weekly intake (EWI) is also elaborated and compared with the provisional tolerable weekly intake (PTWI). As a result, when EWI values are less than the “joint FAO/WHO expert committee on food additives” (JECFA)’s PTWI value for the TEs in question, it is safe to say that no potential health risks exist for individuals who consume the corresponding food items [24].

Trace elements (TEs) persist in the environment for a long time because they are non-biodegradable. TEs found in sediments and soils remain present in the environment until they become eluted. Moreover, these TEs can interact with other elements present in the sediments or the soil which could make them more toxic [25]. Regarding Pb, Cd, Hg, and As, several studies found that the carry-over to muscle, eggs, and milk is usually low when the animal has a standard diet to feed on (the concentration of TEs is below the European Union’s maximum permissible levels). However, an accumulation in certain organs (bones, kidney, liver) was observed with a higher dietary intake of toxic TEs [26]. Moreover, the build-up of TEs differs significantly among different tissues of the animal, as well as between different animal species. Furthermore, it was found that TE accumulation in fish tissues depends largely on their corresponding levels in commercial feed, in water, or in prey. These TEs, such as As and Hg, are found to be dangerous for their carcinogenic potency and they tend to bioaccumulate and build-up in the system [17,25,27]. Since TEs are non-biodegradable and cannot be removed or broken down, organisms may detoxify these TE ions by placing them in certain intracellular granules in insoluble forms for a long storage time, by excreting them in feces, or by hiding them within a protein [25]. 

After a general overview of the TEs and their common characteristics, an introduction of the more commonly present TEs, will be displayed. The TEs which are described in further detail include As, Hg, Pb, Cd, and Cr. The objective is to highlight the environmental exposure of the TEs together with their adverse health effects. Regarding As and according to the WHO, the greatest threat to public health comes from contaminated drinking water, food preparation, and irrigation of food crops [28]. Regarding Hg, public health threat occurs when seafood or rice containing methylmercury are consumed. Regarding Pb and according to the European Commission [29], cereal products and grains, potatoes and leafy vegetables, and tap water are the main source of public health problems in the general European population. For Cd, according to the European Commission [30], cereals, vegetables, nuts, potatoes, and pulses contribute most to the dietary Cd exposure. Regarding Cr, the main human exposure is food. The highest concentrations were found in foods such as meat, fish, cereals, tea, black pepper, and some fruits and vegetables [31].

Table 1 gathers the main toxic elements and the foods in which they were found. We tried to focus on recent studies and on the following TEs: As, Hg, Pb, Cd, and Cr.

### 2.1. Arsenic (As)

Arsenic (As), a metalloid, is a common element found in the Earth’s crust. It is naturally present in several oxidation states (3−, 0, 3+, and 5+) and has different organic and inorganic forms [23]. As is emitted into the atmosphere from both anthropogenic and natural sources. Volcanic activity is the main natural source; also, wind-blown dusts and exudates originating from vegetation contribute to its atmospheric concentration. Moreover, As pollution can originate from aquifers and soils [88]. The total global yearly concentration of As in the air coming from anthropogenic and natural emissions is estimated to be 31 Gg-Ton [89]. A study has also reported that the level of As originating from anthropogenic sources is significantly higher (around 1.6 times) than natural emission [90]. For instance, during the smelting process of Cu and Pb, As is converted to arsenic trioxide (As_2_O_3_) and becomes volatilized. Thus, its concentration amounts to approximately 30% in the flue dust [91]. This As_2_O_3_ is considered as the main form of As recovered from industries [92]. It is also used in insecticides and as wood-preservative [88]. More industrial activities linked to As include wood, gasoline, oil, glass and ceramics manufacturing, electronics, paints, pigments, antifouling agents, metal alloys for electronic circuitry, coal-burning, metallurgical activities, and mining. As a result, such activities lead to environmental contamination with As [92,93]. Furthermore, inorganic As preservatives combined with other substances have been applied for a very long time to various organic objects in natural history museums and in ethnological places [94]. Moreover, As was historically applied as pesticide to treat animal hides; afterwards, tannery wastes high in As are produced. Consequently, this prolonged dumping of wastes led to As contamination of surface and subsurface soils according to a study conducted in Australia [92,95]. However, in 2009, a cancellation request was issued by the U.S. Environmental Protection Agency (EPA) to ban and discontinue the use of arsenical pesticides by the year 2013 [96]. 

**Figure 1:**
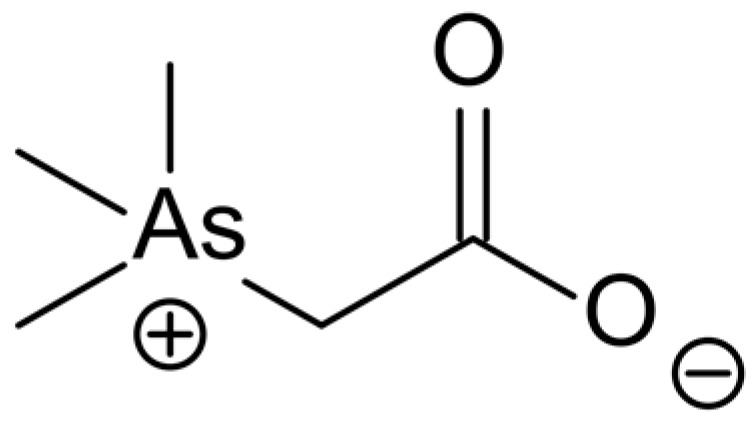
Chemical structure of Arsenobetaine (AB).

Regarding human diet, seafood and fish are the main source of As, where it occurs as organic species (from 1 to 100 mg As/kg) [97] with a total mean content of 0.46 mg per kg of fish flesh to 102.7 mg per kg of edible algae as maximal value [98]. Arsenobetaine (Figure 1), the final As metabolite found in the marine food chain, can bioaccumulate in marine species to a greater or lesser extent. Almost 50 to >95% of the total As is made of arsenobetaine in organs and tissues of gastropods, crustaceans, teleost fish, elasmobranchs, and Polychaeta. Finally, human beings become exposed to As through contaminated food, particularly seafood and drinking water [99].

On the other hand, the toxicity of As is related to several factors, such as the chemical form; in fact, inorganic As is considered more toxic than organic As, and arsenite (As^3+^) is more toxic than arsenate (As^5+^) [100]. Moreover, the order of increasing toxicity for As is represented as organic As species < As(0) < inorganic As species (including As^5+^ < As^3+^ < arsine) [23]. Cases of As poisoning in humans have been reported in India and in Bengal, where thousands of people have manifested symptoms caused by As intoxication after ingesting contaminated food or water [92]. Moreover, As is classified as a carcinogen for animals and humans, even though it has agricultural, industrial, and medicinal uses. According to the United States National Toxicology Program (NTP), the Food and Drug Administration (FDA), and the EPA, As is linked to higher risk of lung, kidneys, liver, and bladder tumors [88]. Moreover, severe intoxication may lead to certain forms of skin cancers [23], and acute As toxicity can cause adverse health effects such as pricking sensation in hands and legs, muscular pain, drowsiness, weakness, and confusion [10].

### 2.2. Mercury (Hg)

Mercury (Hg) is emitted into the water and atmosphere through different anthropogenic activities such as fossil fuel combustion, coal burning, and industrial activity [101]. Moreover, waste incineration, used for the treatment of urban and rural wastes, causes Hg emissions [102]. Elemental mercury (Hg) can bind to sulfur, chlorine, phosphorous, or other elements to form inorganic compounds. Inorganic Hg can also bind to carbon, due to the action of microorganisms, to form organic compounds such as methylmercury (MeHg) and ethylmercury (EtHg). Organic complexes of Hg are seen as the most common and most hazardous of Hg forms; it is frequently found as ethyl-mercury (EtMg) and methyl-mercury (MeHg) [103]. Some forms of organic Hg have a low level of exposure to the public such as dimethylmercury, dialkyl mercurials, and diethylmercury. According to a Food and Drug Administration (FDA) estimation, around 80% to 90% of the Hg would be in the form of MeHg [104]. Therefore, Hg bioaccumulation through the food chain is mostly due to the methylated form of Hg [105]. Mercury contaminated fish are considered the major route of MeHg exposure among humans [106,107,108]. 

Certain forms of organic mercury have different applications. For example, EtHg and MeHg can be used as fungicides [23]. Diethylmercury was used for more than 100 years in small quantities in some specific industrial processes. Phenylmercury was used in the old days in paints; in addition, dialkyl mercurial, such as dimethylmercury, are still used nowadays in certain industrial activities and in the calibration process of certain analytical laboratory machinery. Moreover, dimethylmercury is used as a reference material in laboratories in nuclear magnetic resonance spectroscopy, which has once caused a lethal exposure via dermal contact [109].

Many studies have found a correlation between organic-Hg exposure and higher risks of immunological reactions, neurodevelopmental disorders, and nephrotoxic effects [110]. The toxicity of MeHg differs among gender and age groups; these adverse effects depend upon frequency, exposure time, and susceptibility factors [101]. High exposure levels of MeHg can cause serious neurological damage. Hg usually accumulates in the kidneys and leads to harmful effects, particularly in the proximal tubules [23]. Furthermore, high levels of MeHg exposure have been linked to fetal neurological damage in Minamata and Iraq [101]. Finally, after exposure to MeHg, a latency period of 16 to 38 days is observed before the manifestation of clinical symptoms of poisoning [111]. The cellular uptake of Hg can be passive (e.g., organic methylmercury chloride (MeHgCl) in cell cultures), energy-dependent (e.g., MeHg-cysteine), and active; it all depends on the Hg species [112]. In fact, exposure and inhalation of Hg vapors contribute to elevated concentrations of inorganic Hg in the central nervous system, resulting in neurological adverse effects. The inhaled Hg vapors rapidly cross the pulmonary alveolar membranes due to their lipophilic affinity. Then, Hg enters rapidly into the plasma erythrocytes where it is converted to divalent ionic Hg (Hg^2+^). On the other hand, a part of the inhaled Hg could stay long enough in the bloodstream to finally cross the blood–brain barrier where it undergoes another set of reactions [113]. In addition, the simultaneous incubation of MeHgCl and Thimerosal allows the crossing of Hg, in both ways, through the blood–brain barrier. Hg tends to accumulate slightly in the brain facing compartment, while, when it comes to inorganic HgCl_2_, the cells of the blood–brain barrier tend to move Hg out of the brain [112,114]. Clinically relevant adverse toxic effects of methylmercury (1+) ion (CH_3_Hg^+^) take place in the central nervous system (CNS) and brain. Therefore, many studies have highlighted the mechanisms by which CH_3_Hg^+^ gets access to the CNS, particularly, the way CH_3_Hg^+^ traverses the blood–brain barrier. CH_3_Hg^+^, through mechanisms such as Hg^2+^, is only found as a bound cation in a biological system; in fact, it is found bound to thiols containing biomolecules such as N-acetylcysteine, Cysteine (Cys), gluthathione (GSH), homocysteine, or albumin. Studies using rat cerebrum homogenates found that GSH is the major non-protein thiol conjugated to CH_3_Hg^+^. Later studies suggested that CH_3_Hg-S-Cys are transportable molecules through the blood–brain barrier [115].

### 2.3. Lead (Pb)

Lead (Pb) is a bluish-gray metal that naturally exists in the ground; due to its chemical and physical properties, it is widely used [116]. It is a versatile and ubiquitous metal distributed and metabolized in the environment which leads to an increased human exposure and intake of Pb [117]. 

Exposure to Pb in food and drink has a long history. In Roman times, Pb poisoning was mostly due to the use of Pb in water pipes, wine storage, and earthenware containers [117]. In recent times, human exposure to Pb and its derivatives is mainly due to occupational reasons and other sources such as leaded gasoline [118], synthetic Pb arsenates insecticides that have been used since the 20th century, resulting in contaminated soils with both As and Pb [119], and firearms using Pb bullets [120]. Moreover, studies in sub-Saharan African countries showed that children are at risk from Pb exposure because of the previous use of Pb in gasoline, the poor recycling of electronic waste, and the inappropriate recycling of batteries [116]. Moreover, acidic drinks such as fruit juices constitute a possible exposure route when kept in crystal ware or ceramics containing Pb. In fact, ceramic tableware can be considered as an important source of Pb contamination due to some glazes [121]. Children are in higher risk of adverse effects due to Pb intoxication, which is due to higher Pb dose per body weight unit in children than in adults. Moreover, it was found that the Pb intake is higher in children because they tend to put soiled things in their mouths [117].

After absorption, Pb is carried by the red blood cells to be distributed throughout the organism. Pb is primarily bound to hemoglobin after entering the cell. The hematopoietic system is sensitive to Pb toxicity, which may result in anemia. In fact, a high exposure level can cause damage to almost all organs’ systems, mainly the CNS, blood, and kidneys. On the other hand, a low level of exposure affects biochemical processes and heme synthesis, as well as neurobehavioral and physiological functions [117]. Furthermore, clinical manifestations of Pb poisoning in humans include hearing loss, partial blindness, and various CNS problems (headaches, irritability, insomnia, depression, delirium, etc.) [120].

In addition, chronic Pb exposure can take weeks to months, where it accumulates and leads to toxic adverse effects. Moreover, acute Pb toxicity may arise from high doses in short time exposures [118]. According to the European Food Safety Authority (EFSA), the mean contribution of various food categories to Pb exposure was calculated in 2012 for the general European public [122]. The highest contributors were “grains and grain-based products” at 16.3% followed by “milk and dairy products” at 10.6%, “non-alcoholic beverages” at 10.3%, “vegetables and vegetable products” at 8.4%, “drinking water” at 7.0% and “alcoholic beverages” at 6.7%. 

Interventions were implemented to successfully reduce the level of Pb in food: stopping the use of Pb in glassware such as wine bottles, banning the use of Pb additives in gasoline issued by the EPA’s recommendations, prohibiting the use of Pb solder in food cans in the 1990′s [120], banning the use of Pb in food containers, reducing Pb in water treatment and water distribution systems, and developing analytical quality control program and international monitoring [117]. Unleaded gasoline was known to be the most significant contributor to the decrease of human blood Pb level according to a study conducted in Taiwan [123]. The WHO recommended in their guidelines to reduce the threshold in drinking water from 100 µg/L in 1961 to 50 µg/L in 1977 and finally to 10 µg/L in 1996. Later in 1998, the European Union (EU) proposed that a concentration of 25 µg/L for drinking water should be achieved within 5 years and 10 µg/L within 15 years [124].

### 2.4. Cadmium (Cd)

Cadmium (Cd) is an environmental contaminant causing risks to human health [121]. It can exist in human foodstuffs due to its soil-to-plant transfer [125]. Cd is also widespread in the environment (soil, water, and air) due to several industrial and anthropogenic activities that could cause human exposure to Cd, such as Cd and Ni batteries, use of manure and phosphate fertilizers, mining, recycled electronic waste, non-ferrous metal smelters, and sewage sludge disposal [121,126]. In addition, municipal solid waste is disposed using incineration which releases Cd into the environment due to its low boiling point [127]. Moreover, the use of Cd-plated utensils and galvanized equipment, Cd-based pottery glazes, and Cd-bearing stabilizers in plastics can contribute to Cd contamination of food [121]. In 2013, a study conducted in China estimated the mean dietary exposure to Cd to be 12.8 ± 4.2 μg/day. In addition, three food categories (Vegetables, Seafood and Rice) were found to be the major sources amounting to 86.3% of this overall exposure (Vegetables: 40.2%, Rice: 37.6% and Seafood: 8.5%) [128].

Cd was found to induce hepatic and renal damage in exposed animals [129]. Moreover, human exposure to Cd can cause multiple adverse effects, including hepatic and renal dysfunction and pulmonary oedema. Its cytotoxicity might lead to necrotic or apoptotic occurrences; hence, Cd is classified as a human carcinogen [126]. 

Moreover, Cd accumulates in the organism and has a biological half-life in humans ranging between 10 and 33 years. Thus, the toxicity of Cd usually results from chronic exposure [121]. FAO/WHO recommends the following guidelines for safe intake: PTWI for Cd was fixed at 7 µg/kg body weight [125].

### 2.5. Chromium (Cr)

Chromium (Cr) is widely present in air, soil, and water from both anthropogenic and natural sources. It exists in different oxidation states from −2 to +6. Humans are mostly exposed to chromium in its predominate forms Cr(III) and Cr(VI). The guideline value for total chromium is of 50 μg/L. Even though it is naturally present in the Earth’s crust, chromium mainly comes from anthropogenic sources (more than 70%) [130]. They are used in refineries, nonferrous base metal smelters, urban storm water runoff, leather tanning industries, paper mills, manufacturing of catalysts, paints, pigments and fungicides, ceramic and glass industry, photography, chrome alloy and chromium metal production, and chrome plating [131,132]. Naturally, it exists in small amounts in soils and rocks where it can be released through erosion processes and weathering [130].

In the environment, Cr(III) mostly occurs as chromium hydroxide (Cr(OH)_n_ ^(3−n)+^) and Cr(VI) as chromic acid (HCrO_4_^–^) or as chromate ion (CrO_4_^2–^). In soil, Cr(III) is the predominant form because Cr(VI) can be reduced by organic matter to Cr(III). Cr(VI) salts are more soluble than Cr(III) salts in water, which makes Cr(VI) more mobile. It should be noted that Cr(VI) may penetrate cell membrane but Cr(III) may not, making Cr(VI) the more dangerous form. In the atmosphere, chromium is present as aerosols and might be removed by wet or dry deposition [130]. 

Chromium is not thought to bioaccumulate in the aquatic food chain [133]. Cr(VI) is transformed to Cr(III) when absorbed by fish [134]. Moreover, studies show that Cr has a low mobility when moving from the roots to aerial parts of the plants [135], and the transfer from soil to plant is not well investigated [130].

Cr(III) is considered by the United States Institute of Medicine to be an essential nutrient and its adequate intake determined to be 20–45 μg/day [136]. However, there were no evidence of deficiency in humans; thus, this view is found to be equivocal and no beneficial effect of supplementation was demonstrated [130]. 

Chromium intoxication in humans can cause severe gastrointestinal problems, liver, kidney, and lung damage, as well as cardiovascular collapse brought on by severe hypovolemia. In various case studies including both adults and children, deaths have been linked to the consumption of Cr(VI), at levels ranging from 4.1 to 357 mg/kg bw/day. A deadly dose of potassium dichromate (K_2_Cr_2_O_7_) is regarded as being around 1 g. Moreover, the skin showed eczematoid lesions, scarlatinoid, and pustular eruptions, macular erythema when in contact with soluble Cr compounds. Long term exposure result in systemic, neurological, reproductive, developmental, and immunological effects through the genotoxicity and carcinogenicity of Cr(VI) [131].

## 3. Analysis Strategy

Despite recent developments, sample preparation is still to be improved in order to meet the same high requirements as the instrumental procedures needed for analyte determination [137,138]. For the inductively coupled plasma mass spectrometry (ICP-MS) determination of As, Cd, and Pb, there are six primary digestion processes that are frequently reported in various studies: electro-thermal vaporization, dry ashing in a standard oven, oxygen combustion, acid digestion in an open vessel, microwave digestion in a sealed vessel, and microwave digestion [137]. Currently, most labs and enterprises still prepare samples for instrument analysis via dry ashing and wet digestion [137].

Solid phase extraction (SPE), one of several pre-concentration and/or separation techniques, is carried out using batch and column techniques. Elements or species of elements of interest are retained by sorption on various solid phases and eluted with acids or other reagents. There are multiple benefits to using the solid phase extraction approach to pre-concentrate trace elements from various materials [139].

Flame atomic absorption spectrometry (FAAS), inductively coupled plasma atomic emission spectrometry (ICP-AES), inductively coupled plasma mass spectrometry (ICP-MS), and X-ray spectrometry are often employed for trace element analysis in food samples [140].

Before choosing a technique, one should consider factors such as sensitivity and detection limit, analytical precision, concentration range, analytical interferences, cost, experienced staff, laboratory size, laboratory specialization, and capacity to control sources of contamination [141].

Different calibration strategies, including external standard calibration (EC), matrix-matched calibration (MMC), internal standardization (IS), and standard additions (SA), are used to overcome matrix effects and improve accuracy and precision in trace element analysis of all types of samples using instrumental spectrochemical methods [142]. It is crucial to regularly analyze reference materials to ensure the quality of the results and harmonize them. Ideally, they should be approved by a recognized international organization and match the sample in terms of matrix and element concentration [141]. Furthermore, the validation of analytical techniques to identify both essential and hazardous components in food is crucial for producing accurate results [143].

**Figure 2:**
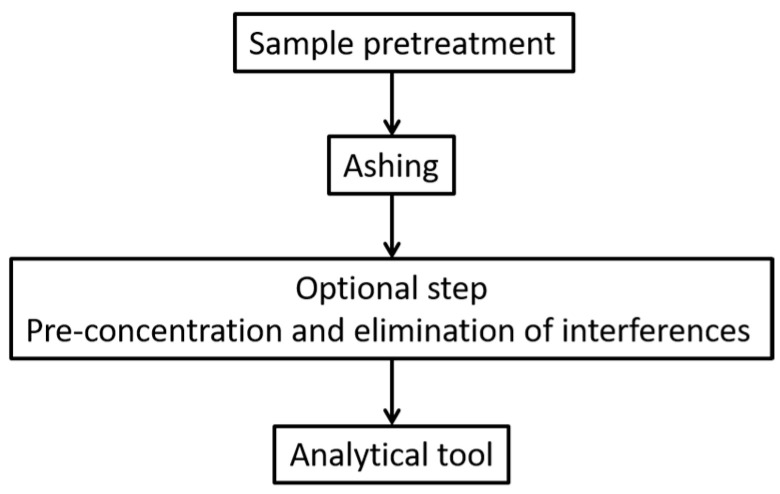
The overall analytical strategy in trace element analysis.

The general route for trace element analysis is given in Figure 2. It begins by sample pretreatment where issues such as sampling, particle size reduction of the sample, precautions to avoid sample loss, and contamination are addressed. The second step is to remove the organic matter by ashing the sample. The operator has to choose between wet or dry ashing using open vessels or microwave devices. In wet ashing, the choice of the acid or the acid mixture is important to have a complete dissolution of the analyte. The third step of pre-concentration is needed when low amounts of trace element are present in the food matrix below the detection limit of the analytical tool. Sometimes, an elimination of interferences step is needed depending on the choice of the analytical tool and the type of interferences. The final step is the adequate choice of the analytical methods; in this case, the limit of detection, the type of interferences, and the availability of the analytical device will help the user in his choice.

### 3.1. Sample Pretreatment

The sampling process for trace element analysis involves several steps, starting with the sampling of the source material and ending with the final extraction of the analytical subsample. Beyond the initial sampling, each level of sampling before analysis is carried out with the aim of lowering the amount of material being processed while keeping the subsample representivity (i.e., attempting to maintain a strong correlation between the physical and chemical characteristics of the subsamples and that of the primary sample) [144].

The reduction of particle size can be accomplished in several ways, such as mechanical grinding or crushing, cutting in a Wiley or hammer mill, abrasion in a cyclonic mill, or crushing in a ball mill. The sample will be more homogeneous if well grinded [145]. The three common forms of the sample material is sent are solids, solutions made directly from wet-ashed material, and solutions made from dissolved dry-ashed material [144].

Because of their high fat, moisture, or sugar content, animal products (meats), syrups, and spices need to be handled carefully before ashing to prevent sample loss due to spattering, bubbling, or foaming. After drying and fat extraction, a sample may be ashed. To prevent the risk of solvent ignition or explosion, fat-extracted samples should not be heated until all combustible extraction solvents (hexane, ether, etc.) are entirely evaporated [146].

During laboratory processing, a sample may lose material. A fine residue, ash, is formed after a sample is dry ashed. Any air flow across the sample can easily carry the residue’s tiny particles. A stream of gas is passed over the sample as it is being heated to help in combustion, or air flows are created by temperature variations (such as opening the furnace when it is hot). Under certain circumstances, some elements can become volatile (e.g., heat, grinding, strong oxidizers). For example, mercury is relatively volatile; the vapor pressure for Hg is 0.26 Pa at 20 °C (PubChem data). To stop the element of interest from volatilizing, special preparation techniques should be applied. Sample materials may lose certain components because of contact with the container. These losses could be significant. The use of pre-treated glassware with an established hydrated layer can reduce losses caused by adsorption in glass and plastic vessels [147].

Potential sources of trace element contamination include skin, perspiration, dust, tubes and glassware, vessel type, anticoagulants, chemicals used during the digestion process, and homogenization or grinding techniques used for solid samples. As a result, some suggestions include using powder-free plastic gloves, ultra-pure chemicals, and highly purified water in addition to acid cleaning of laboratory glassware and plastic containers and maintaining clean lab environments [141].

In order to eliminate or reduce physical interferences, some techniques are used such as dilution of the samples, matrix matching of the standard and blank with the sample, use of internal standards, chemical separation, and standard addition method [141].

### 3.2. Sample Digestion 

Microwave digestion is used in both dry and wet mineralization. Microwave devices can speed up the ashing process in both scenarios, although sample amount might be a constraint. The main goal of sample digestion is to change the sample’s shape into one that can be chemically analyzed [148]. To avoid the production of smoke during the heating process, high-fat goods such as meats may need to be dried and the fat drained before ashing [146].

Mineral contamination from the environment or from the grinding apparatus is a potential concern if ashing is employed as a pre-processing step for mineral assessments and may necessitate the use of sample blanks [146]. Muffle furnaces are frequently employed in traditional dry ashing techniques to burn samples with high levels of organic materials in an open system. As a result, samples can be easily contaminated by the environment, and components can be volatilized. The addition of ashing aids is a popular technique for preventing elemental volatilization. Ashing aids, on the other hand, will raise the cost of the reagents as well as the possibility of contamination [137]. Additionally, compared to traditional open wet digestion, dry ashing necessitates less chemicals [137].

Dry ashing will result in the loss of volatile components, whereas wet oxidation barely causes any volatilization. Wet or dry ashing can be used depending on the specific elements being investigated and the elemental analyses needed. Some micro- and highly volatile components will call for specialized tools and methods [146]. Additionally, it is important to consider how the reagent interacts with the analytes because in open digestion systems, some analytes may precipitate or even disappear due to the production of volatile molecules. For instance, caution should be used when using hydrofluoric acid for digestion in open systems when the analytical aim is the measurement of boron in glass since the analyte may generate BF_3_ species that may be lost by volatilization [149]. Additionally, the length of the extraction process and the total amount of reagent used are crucial for acid digestion methods [150].

The method of choice depends on the use of ash after it is obtained as well as by the restrictions in terms of price, duration, and sample size [146]. Inefficient and expensive equipment are unsuitable for daily large sample analysis [137].

#### 3.2.1. Dry Ashing: Conventional Oven

Ashing is conducted in an oven at temperatures ranging between 475 and 600 °C or even higher [145]. The inorganic residue (i.e., ash) is oxidized by air oxygen, and primarily consists of metal oxides as well as non-volatile sulfates, phosphates, and silicates [151]. Typically, the ash residue is dissolved in HNO_3_ or HCl solutions and then diluted with deionized water [145].

Although this process is rather straightforward, there are significant applications where it cannot be used since some elements may be volatilized and lost entirely or partially. Higher decomposition temperatures result in more severe volatilization losses [151]. The volatile elements As, B, Cd, Cr, Cu, Fe, Pb, Hg, Ni, P, V, and Zn are at risk of being lost [146]. In addition, complex metal oxides are produced when dry ashing and acid extraction are combined (i.e., refractories which are very difficult to dissolve). When carbonaceous materials are thermally pre-treated, it is possible for difficult-to-dissolve phases (such carbon compounds such as carbides) to form [152]. The time needed (12–18 h or overnight, depending on the sample weight and type) and the expensive equipment are also disadvantages.

The benefits of conventional dry ashing include that it is a safe and affordable method, that it requires fewer samples than other methods, that it does not call for acids or other additional reagents or blank subtractions, that it has a high sample recovery rate, and that once ignition starts, only minimal attention is required [146].

#### 3.2.2. Dry Ashing: Microwave Oven

Microwave muffle furnaces can ash samples in around 20 min, cutting down analysis time by as much as 97% compared to normal dry ashing in a muffle furnace, which could take many hours. Temperatures as high as 1200 °C can be reached in microwave muffle furnaces. These systems can be set to automatically warm up and cool down using a variety of techniques [146,153]. Additionally, digestion is usually carried out under high pressure in enclosed vessels heated by microwave energy. This enables the sample to decompose more quickly while minimizing external contamination and volatile chemical losses [141]. Microwave furnaces may not be able to contain as many samples as the traditional ones, but they can process a lot more samples in the same amount of time [146].

#### 3.2.3. Wet Ashing: Open Vessel

A crucial step before analyzing trace elements is sample pre-treatment. To digest organic material and transform the analyte into an appropriate form for analysis, it is required to choose and optimize the digestion process to remove matrix effects and other interference factors.

Wet oxidation or wet digestion are other names for wet ashing. It is mainly applied to prepare for mineral analyses [146]. Strong oxidizing acids can be added to the sample and heated to facilitate wet digestion procedures by breaking down the sample’s organic components. Mixtures of nitric and perchloric acid (HNO_3_-HClO_4_), sulfuric acid (HNO_3_-H_2_SO_4_), hydrochloric acid (HNO_3_-HCl), and hydrogen peroxide (HNO_3_-H_2_O_2_) are frequently used as oxidizing agents. The maximal digesting temperatures range from 122–338 °C at ambient-pressure; they are constrained by the acid or acid mixture’s used [148].

Although hydrochloric acid (HCl) is a non-oxidizing acid when used alone, it becomes a strong oxidizing agent when combined with nitric acid (aqua regia) (HCl:HNO_3_ 1:3) [154]. Since HCl is a weak reducing acid, it is rarely utilized alone to break down organic compounds. 

Perchloric acid (HClO_4_) has potent oxidizing and dehydrating capabilities and will strongly react with organic substances especially when used concentrated and hot. It is therefore advisable to pre-treat organic samples containing organic material using HNO_3_ or HNO_3_-HClO_4_ solutions in order to avoid violent reactions. When anhydrous, several HClO_4_ salts can spontaneously catch fire. HClO_4_ has a high boiling point of 203% and is used at 72.4%.

Concentrated sulfuric acid (H_2_SO_4_) has the highest boiling point of the mineral acids (338 °C for the 98.3% acid). They also have dehydrating and mildly oxidizing abilities.

The process of wet ashing has many benefits. Due to the lower temperatures and short oxidation duration, trace elements usually remain in solution with little to no loss due to volatilization [146]. According to Kingston and Jassie, a wet digestion in an open vessel takes between one to two hours to complete, but it may take longer under certain circumstances [154]. Wet ashing has certain drawbacks: it takes virtually constant operator attention and corrosive reagents; high acid consumption rates are necessary, which may create interferences and environmental damage [155].

During the digesting process, contamination can occur from the reagents, the vessel materials, and the environment. This could be an unanticipated obstacle in sample analysis which can produce erroneous results [148]. Moreover, only a few samples can be processed at once [146].

Even though wet digestion with perchloric acid is an AOAC process (e.g., AOAC Method 975.03), many analytical laboratories use a mixture of nitric acid with sulfuric acid, hydrogen peroxide, or hydrochloric acid instead of perchloric acid in wet ashing. This is because working with perchloric acid can result in extremely dangerous explosive peroxide by-products [146].

#### 3.2.4. Wet Ashing: Microwave

It is safe to conduct microwave wet ashing (acid digestion) in either an open- or closed-vessel microwave system. Acids may be heated over their boiling temperatures because of the closed vessels’ capacity to endure higher pressures (some vessels may take up to 1500 psi). Nitric acid is capable of reaching a temperature of 240 °C when contained in closed containers created especially for high temperature/high pressure reactions.

Up to 40 samples can be processed at once using closed-vessel microwave digestion systems. Samples are placed in vessels with the right amount of acid. More samples can be processed at once using this technique than with the conventional techniques, which increases throughput. Normal digestions last under 30 min. Vessels must cool down before being opened because pressure is created with heating. For larger sample quantities (up to 10 g), open-vessel digesting methods are mostly used. The apparatus is turned on, acid is supplied, and the vapor containment device eliminates reaction-related emissions [146].

When compared to conventional methods, microwave digestion procedures are preferred due to their advantages of short digestion time, low acid usage, and high extraction efficiency [150].

## 4. Pre-Concentration and Precautions

### 4.1. Pre-Concentration

In situations where the element to be analyzed is present in the sample at a level below or close to the detection limit of the instrument, several procedures have been developed for pre-concentration of trace elements. These steps are intended to reduce the detection thresholds and bring the analyte concentration inside the detector’s dynamic range [140,141].

A solid-phase extraction (SPE) has frequently been combined with flame atomic absorption spectrometer (FAAS) due to the high enrichment factor, high recovery, low cost, minimal consumption of organic solvents, and the possibility to combine with diverse detection techniques [140]. In addition to removing organic matrix interference and ensuring sample homogeneity, through destroying the sample matrix, particularly the organic components, it enables pre-concentration of the elements, allowing for improved precision and accuracy in the analytical results [141].

For trace analysis of lead, cadmium, copper, cobalt, chromium, nickel, tin, and gossamer blue, for the pre-concentration techniques such as solvent extraction, ion exchange resins were frequently used for the separation and pre-concentration of radionuclides from environmental samples due to their low cost and wide application ranges [140,156].

For samples pre-concentration, liquid–liquid extraction, which transfers analyte from the aqueous sample to a water immiscible solvent, is frequently used. It is common practice to prepare samples using cloud point extraction (CPE), which works similarly to liquid–liquid extraction by moving the analyte from the aqueous sample to a water immiscible solvent [140].

### 4.2. Precautions

In low-level operations, contamination from radiochemicals in reagents is particularly problematic. It is important to avoid such contaminations as much as possible. Cleaning glassware and equipment is necessary in sample processing. Glassware should be frequently checked for nicks, cracks, and other damages and discarded if they are found to be present. Glassware contamination should be checked using blanks and screening. It is advised to utilize brand-new or disposable labware or containers whenever possible. The cost of using disposable plastic centrifuge tubes is usually cheaper than that of using glass tubes, which must be cleaned after each use. To lessen the risk of contamination while using non-disposable containers or labware, fresh materials are used for every new project. Frequent rinsing with a diluted nitric acid solution can help keep glassware clean. It was found that washing with powerful mineral acids did not effectively remove nuclides that had adhered to the walls of plastic containers, whereas the use brushes in the cleaning procedure successfully removed adsorbed nuclides [147].

## 5. Analytical Methods

### 5.1. Introduction

The development of the transistor in 1947 was crucial in transforming instrumental approaches to trace analysis. Ion selective electrodes, various electrochemical sensors, and combinations of electrochemical and optical techniques are only a few of the directions electroanalytical chemistry has taken since 1950 [157]. Additionally, Varian created the first combination spectrophotometer, known as the Cary 11, in 1947. The first optical emission spectrometers with photomultiplier tubes as detectors were created in 1947–1948 [158].

Strangely, the atomic absorption technique did not become popular until 1955. The subsequent dramatic rise was largely caused by Walsh’s hollow cathode tube. However, another aspect was the huge increase in demand for trace analytical data during the 1950s and the 1960s. Alkemade proposed the concept of atomic fluorescence spectroscopy in 1962, and Winefordner first applied it analytically. Winefordner noted in 1976 that despite the method’s benefits over atomic absorption, it had not gained much traction. The causes why are not clear. Although the absence of commercial instruments could be a factor, atomic absorption techniques’ enormous popularity is more likely to be the reason.

Inductively coupled plasma (ICP) spectroscopy was introduced by Fassel and Greenfield in 1964 and is the most popular of today’s emission spectrochemical techniques due to its commercial availability and suitability for very sensitive multi-element analyses [157]. Since its commercial debut in the mid-1970s, inductively coupled plasma-optical emission spectrometry (ICP-OES), also referred to as inductively coupled plasma-atomic emission spectrometry (ICP-AES), has grown quickly in popularity for a variety of applications involving the determination of trace elements in different samples [159]. The 1980s witnessed the commercial introduction of inductively coupled plasma mass spectrometry (ICP-MS), which has since expanded quickly [160].

Over the past few decades, the performance of analytical apparatus for chemical analysis has improved remarkably. Flame atomic absorption spectrometry (FAAS), graphite furnace atomic absorption spectrometry (GFAAS), inductively coupled plasma atomic emission spectrometry (ICP-AES), and inductively coupled plasma mass spectrometry (ICP-MS) are the most used methods today to analyze trace elements in food samples.

### 5.2. Atomic Absorption Spectroscopy (AAS)

**Figure 3:**
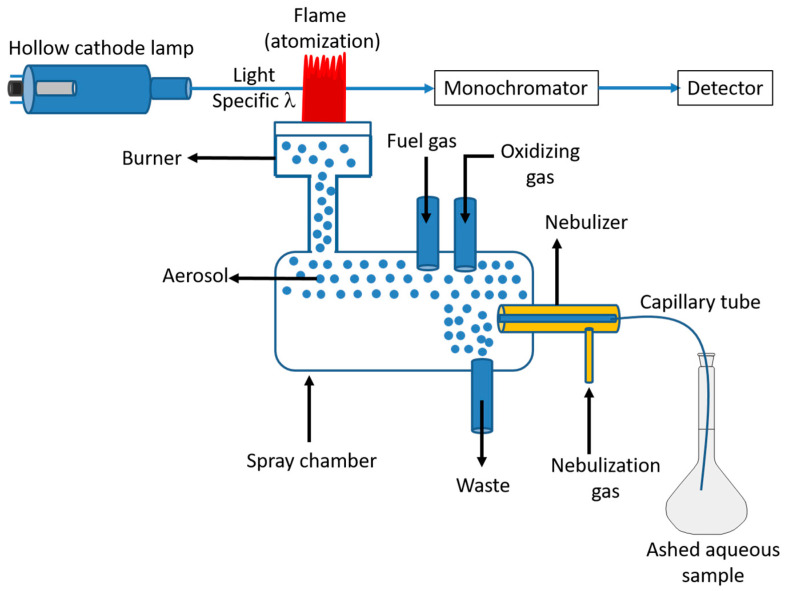
Flame atomic absorption spectrometry (FAAS) principle.

Atomic absorption uses two fundamental types of light sources, the most popular of which is the hollow cathode lamp (HCL). The cathode of this lamp has the specific analyte metal plated on it. An electric current inside the lamp ionizes the inert filler gas (neon or argon), and the ions are subsequently drawn to the cathode. The metal ions that are deposited on the cathode are excited by the inert gas ions that bombard it. The emission of radiation with wavelengths resembling those of the analyte is caused by this stimulation of the metal. Through an entrance slit, light of all wavelengths enters the monochromator. A prism or, more frequently, a diffraction grating is then used to separate the light into its individual wavelengths. Only light of the desired wavelength goes through the exit slit to the detector by adjusting the position of this dispersing device [161]. The detector measures the absorbance, which is proportional to the concentration. In this method, a nebulizer and spray chamber assembly are used to inject a sample [140]. The Venturi effect causes the sample to be pulled up the intake capillary. The air-acetylene burner with a 10 cm length is the flame cell that is most frequently employed. The flame produced burns at a temperature of about 2300 °C, though the precise temperature will vary depending on the fuel/air ratio. Inside the flame, the molecules are dissociated and transformed into free atoms. The food sample should be ashed and solubilized in an acidic aqueous medium before it is introduced in the nebulizer (Figure 3).

AAS is typically the method of choice for analysts when concentrations are in the range of one part per million (ppm) or even as low as several parts per billion (ppb) with less than four different TEs to be measured [162].

Due to its relative simplicity and low cost of equipment, flame FAAS is one of the most widely used methods for determining trace metal ions. FAAS is also the method of choice when only one or small number of elements need to be identified in a sample [163].

Another variant of AAS is graphite furnace atomic absorption spectrometry (GFAAS). It involves vaporizing a sample and passing the resulting vapor through a graphite furnace, which is a small, cylindrical chamber made of graphite. The sample vapor is then exposed to a beam of light, which excites the atoms in the sample. The light that is absorbed by the atoms is measured by a detector, and the absorption intensity is used to determine the concentration of the element in the sample. As a result, many parts of the equipment needed for GFAAS and FAAS are the same. Both methods require the same light source, background correction system, monochromator or polychromator line isolation device, photomultiplier or charge-coupled device detector, and readout system [161]. The main difference lies in the atomization of the sample which is conducted in a graphite furnace for GFAAS and in an acetylene/air flame for FAAS.

The most appealing for the direct analysis of solid materials was found to be GFAAS, primarily due to the lack of nebulizer system, which makes it easier to add solid samples to the atomizer. The large sample residence time in the GFAAS atomizer also enables the atomization of particles of any size or volatility. Additionally, it has relatively low limits of detection, which is particularly desired in trace analysis. All of these characteristics have made solid analysis by GFAAS an acknowledged methodology and a very practical way to determine trace elements in a variety of solid materials [164]. The utilization of substantially higher atomization temperatures, up to 3000 K, distinguishes GFAAS from FAAS. FAAS can be performed without the necessity of previous pre-concentration of analytes and is commonly used for determining low concentrations of elements (e.g., Al, Ca, Co, Cr, As, Cd, Cu, Fe, Mn, Ni, Pb, Zn) [140,163].

Numerous spectroscopic techniques, including atomic spectroscopic methods such as atomic absorption spectroscopy (AAS), are used for wine and grape analyses [165]. Additionally, a study was conducted to analyze dairy products (cheese) using AAS for minerals such as salt [166]. In another study, beer samples’ Cu, Mn, Pb, and Zn concentrations were determined using thermospray flame furnace atomic absorption spectrometry (TS-FF-AAS) [167]. In other studies, the concentrations of Al, Cd, Pb, Cu, Ni, Mo, Mg, and Zn as well as the concentrations of Ca, K, Mg, Zn, and Fe in grape leaves were assessed using atomic absorption spectrometry [168,169].

### 5.3. Inductively Coupled Plasma Atomic Emission Spectroscopy (ICP-AES)

**Figure 4:**
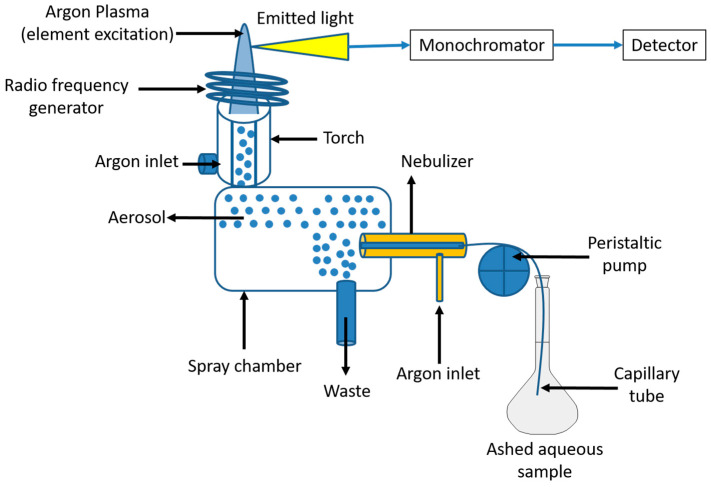
Inductively coupled plasma atomic Emission spectroscopy (ICP-AES) principle.

Inductively coupled plasma atomic emission spectroscopy ICP-AES, also known as inductively coupled plasma optical emission spectrometry (ICP-OES), permits an accurate identification of trace elements [140]. An electrically conducting gaseous mixture of argon, argon ions, and electrons is an inductively coupled plasma (Figure 4). A stream of argon gas that has been stimulated by a powerful radio frequency field is used to create plasma. This causes the flowing argon to be highly atomized and ionized, which produces an excitation temperature of 7000–10,000 °K [67] that is used to excite the analyte present in the sample [140]. Peristaltic pumps are used as sample introduction systems in inductively coupled plasma ICP spectrometry [161]. The liquid sample is first injected into the plasma torch as an aerosol mixture with argon, where the high temperature effectively produces desolvation, volatilization, atomization, excitation, and ionization of the sample [140,141]. The collisions between the neutral argon atoms and the charged particles result in the creation of a stable plasma. The moment the sample is added to the plasma, it is immediately broken down into charged ions by collisions with electrons and charged ions. Different molecules disintegrate into their corresponding atoms, which then lose electrons and repeatedly mix in the plasma. Electromagnetic radiation with a specific element’s characteristic wavelengths is emitted by excited atoms. When detected by a photomultiplier or a semiconductor detector, the intensity of this emission serves as an indicator of the element’s concentration within the sample.

Even though ICP-AES’s detection limits are comparable to those of FAAS, it has a far wider dynamic range and can pick up more components at once. On the other hand, ICP-AES experiences several interferences and is significantly more expensive than AAS methods. Signal intensities produced from analytical elements may be affected because matrix components from sample solution alter the state of ICP. The limits of detections may disqualify this method from direct investigation of extremely low element levels. Therefore, a successful pre-concentration phase is necessary before detection with ICP-AES, such as in the previously discussed FAAS analysis [140]. 

As examples of its application, ICP-AES was used to assess the content of trace elements in milk powder and infant food samples sold in Iran. Trace elements’ concentrations (Al, As, Cd, Cr, Co, Fe, Hg, Pb, Ni, Zn, and Se) were assessed [170]. ICP-AES was also employed for the analysis of newborn formula [149].

### 5.4. Inductively Coupled Plasma Mass Spectrometry (ICP-MS)

**Figure 5:**
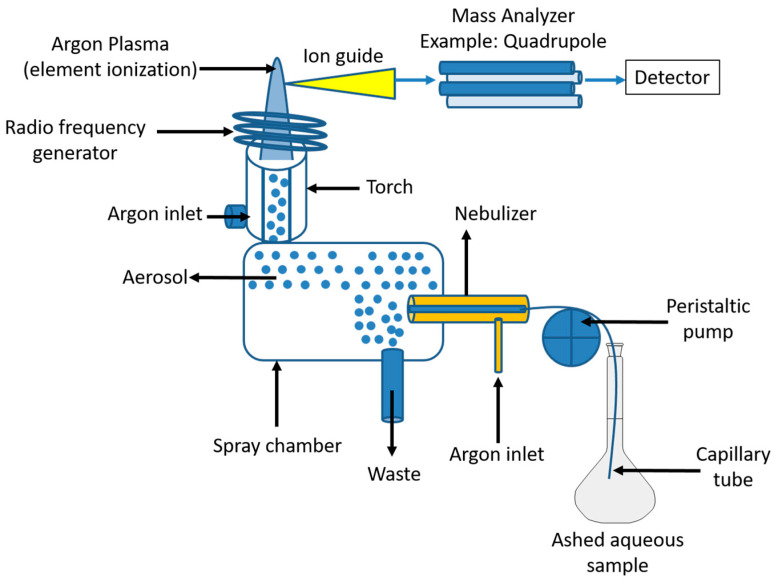
Inductively coupled plasma mass spectrometry (ICP-MS) principle.

In routine multielement determination at trace and ultratrace levels in liquid samples with various matrix compositions, ICP-MS is commonly used. The limits of detection of analytes can be improved to the level of sub-g/L or even to sub-pg/L by the employment of separation and enrichment procedures. Most elemental analyses using ICP-MS that have been reported in the literature deal with easily accessible materials [156]. As in the ICP-AES method, the analyte is ionized in an argon plasma in the ICP-MS method (Figure 5). Nebulized water matrix and chemical compounds evaporate in the plasma, where they are broken down into their atomic components and ionized into positively single-charged ions. Ions are removed from the argon plasma into a mass analyzer such as time of flight (TOF-MS), double focusing sector field (SF-MS), and quadrupole (Q-MS) analyzers. In mass analyzer, ions are separated according to their mass-to-charge ratio or energy-to-charge ratio in double focusing SF instruments [140]. A detector (electron multiplier or Faraday cup) counts the ions that come out of the mass analyzer in the end. Liquid solution nebulization is the most popular and cost-effective among the many sample introduction methods created for ICP-MS [156].

ICP-MS technique has a wide range of applications; it is commonly used in food and industrial analysis and others because it has excellent sensitivity, very low limit of detection, isotopic information, multi-elemental analysis, and throughput [126]. Despite its problems with atomic and molecular isobaric interferences and multi-elemental interferences as well as high cost [140], ICP-MS was recently used to determine Cd, Pb, and As in salted food [171], Cd, Hg, Mn, Pb, and Sb in rice-based products [172] and As, Cd, Cr, and Pb in peanuts [173].

### 5.5. X-ray Spectrometry

**Figure 6:**
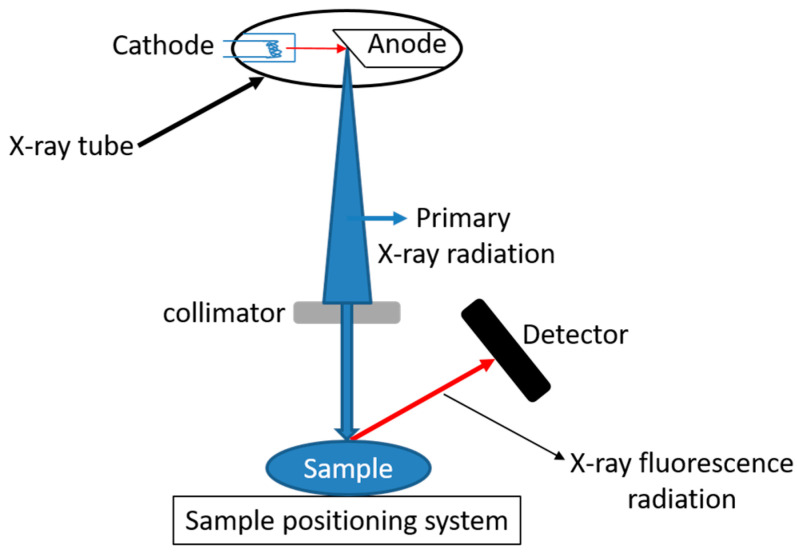
X-ray Fluorescence spectroscopy (XRF) principle.

In X-ray fluorescence (XRF), the sample is irradiated with a beam of primary X-radiations generated by an X-ray tube. The collimator limits the cross-section of the primary beam and allows the excitation of a defined spot of the sample. Due to this excitation, fluorescence radiation is emitted. An energy dispersive detector measures the energy distribution of the fluorescence radiation. The latter is characteristic of the elements present in the sample and can be used to identify the elements and determine their relative concentrations (Figure 6).

Due to its non-destructive and continuous readings, X-ray fluorescence spectroscopy (XRF) has developed into a reliable technique for acquiring high resolution elemental records. For several of the examined items, the optimized operating conditions improve the minimal detection limits and detection efficiency. X-ray fluorescence spectrometry has advantages over other multi-element techniques—for example, ICP-MS/ICP-OES—such as limited preparation needed for solid samples, non-destructive analysis, increased overall speed, reduced creation of hazardous waste and low running costs [174]. Total-reflection XRF (TXRF) uses a radiation that is incident on the sample at an smaller angle than the critical one and completely reflects it back [163]. X-ray fluorescence (XRF) spectrometers are frequently used to identify elements with atomic numbers ranging from 4 (beryllium) to 92 (uranium) at concentrations ranging from 0.1 µg/g to high percentage levels. 

By using Bragg diffraction, X-ray wave phenomena, or energy-dispersive systems, these elements’ distinctive X-ray lines can be identified either sequentially or simultaneously with wavelength-dispersive spectrometers. Increased background effects may result from coherent and incoherent primary X-ray scattering in the sample, and serious matrix effects could also result from the distinctive secondary X-rays’ matrix-dependent absorption.

Since XRF techniques are frequently employed, techniques for addressing matrix effects such as fundamental parameters have been developed. In the 1990s, polarized X-ray fluorescence (PXRF) and total reflection X-ray fluorescence (TXRF) spectrometers, which have significantly improved peak to background ratios, were also developed. An electron from the inner orbitals of the target atoms may be ejected if the target is exposed to photons or charged particles (electrons or ions) with energies greater than the binding energy of the bound inner electrons [175]. High-definition X-ray fluorescence (HDXRF) was recently used to determine As, Cd, Ni, Pb, Sn, and Zn in scallops [176]. 

## 6. Choice of Assay Method

It is obvious that no one digestive method can be used to determine all the components [148]. Numerous samples (biological, clinical, environmental, etc.) have complex matrices containing inorganic chemicals and significant concentrations of soluble solids (i.e., salts of Ca, K, Na, Mg, chlorides, phosphates, sulfates). A study of these types of materials presents numerous challenges such as sample introduction, non-spectral interferences, and spectral interferences in measurements by inductively linked plasma atomic emission, mass spectrometry, and atomic absorption spectroscopic methods. Therefore, samples must be mineralized or at the very least diluted to reduce the quantity of concomitant compounds before analysis in order to remove the organic matter [140].

The range of the analyte concentrations in the sample is crucial information since the sample bulk and digest dilution at the end may be directly connected to the limit of detection. When the amount of analyte in digests is very low, digestion systems that use higher sample masses should be taken into consideration. This is frequently the case with harmful TEs (such As, Cd, Hg, and Pb), which may be present as contaminants in food, drugs, and other materials [149]. Before choosing, one should consider factors such as sensitivity and detection limit, analytical precision, analytical interferences, cost, laboratory size, laboratory specialization, and ability to control sources of contamination, as well as the analytical technique’s multi-element capability [141]. 

From a practical point of view, the user can use Table 2 in order to do a rapid choice of the analysis method. The main parameters of choice are the number of elements the experimenter wants to determine simultaneously, the concentration level in the analyzed sample, the sample number, and the sample volume.

One of the main criteria for choosing an analytical method is the limit of detection. In Table 3, the theoretical detection limits for the most studied essential and toxic elements are gathered. While AAS and XRF allow the determination of ppm level, ICP-OES and GFAAS allow the determination of the ppb level, while only by using ICP-MS it is possible to determine very low concentrations in the ppt range. Another important criterion is the ability of the method to analyze several elements simultaneously. AAS is a limited technique in terms of multielement analysis, but it is reasonably priced and requires less maintenance than other techniques.

## 7. Conclusions and Future Prospects

The initial stage in preparing a food sample for a particular elemental analysis, whether for vital nutrients or for extremely dangerous trace elements, is ashing. Ash content can be significant from a dietary, toxicological, and food quality perspective. It is common practice to do digestion in closed vessels under high pressure utilizing acids and microwave energy. Taking precautions to prevent trace-element contamination during sample collection, storage, and processing is important. 

In situations where the element to be analyzed is present in the sample at a level below or close to the detection limit of the instrument, several procedures have been developed for pre-concentration of trace elements such as SPE, liquid–liquid extraction, and ion exchange resins.

After the sample undergoes the initial preparations, analytical techniques are applied to detect and quantify the trace elements in question. It may be crucial to measure very low concentrations as a sign of exposure to metal contaminants. In this situation, delicate instruments and strict attention against sample contamination are required. Techniques such as AAS, ICP-AES, ICP-MS, and X-ray spectrometry are applied. Certain criteria are necessary for choosing an analytical method such as the limit of detection, cost, or the ability of the method to analyze several elements simultaneously. While AAS and XRF allow the determination of ppm level, ICP-OES and GFAAS allow the determination of the ppb level, while only by using ICP-MS it is possible to determine very low concentrations in the ppt range. AAS is a limited technique in terms of multielement analysis, but remains the most used method for TE analysis thanks to its affordable use and maintenance in comparison with the others tools. 

The methods described in this review concern the determination of total amounts of trace elements. However, trace elements are present in different forms in foods. The current challenge is to develop methods capable of determining the different forms (i.e., speciation). The toxicity and the bioavailability of the elements depend on their form. What makes speciation difficult is the fact that it depends on the food matrix and on the processing.

The main challenges in the determination of trace elements in food samples are: 

(i) Sample preparation: Trace elements are present in very small concentrations in food, so it is important to have an efficient and reliable method for sample preparation that ensures that the trace elements are not lost or contaminated during this step. The use of a suitable method of ashing, a suitable digestion acid, and pure acids reduces errors in the sample preparation step;

(ii) The presence of interferences: The presence of other elements in the food sample can interfere with the determination of trace elements, leading to inaccurate results. Careful sample preparation and analytical techniques that can selectively detect the trace elements of interest can help to minimize interferences; 

(iii) Detection limits: Trace elements are present in very low concentrations, so it can be challenging to detect them using conventional analytical techniques. Techniques with high sensitivity, such as inductively coupled plasma mass spectrometry (ICP-MS), are commonly used to detect ultratrace elements in the ppb level;

(iv) Matrix effects: The sample matrix can affect the efficiency of the analytical technique and the accuracy of the results. The standard addition method in one of the main methods used that takes into account the matrix effect.

Trace element have been determined in various types of food samples. Quality control measurements such as blank, standard addition, spike recovery, replicate analysis, and the use of reference materials remain crucial to guaranteeing the results’ accuracy.

## Figures and Tables

**Table 1:** Concentration ranges in some foods for As, Hg, Pb, Cd, and Cr in different countries of the world.

TE/Food	Year *	Country	Levels **	Reference
As/Drinking Water	2017	Mexico	7–600 µg/L	[32]
	2017–2019	India	0.01–732 μg/L	[33]
	2018	Iran	0.01–12.5 µg/L	[34]
	2019	Pakistan	3.25–184 µg/L	[35]
	2022	Some areas of the world	50–100 µg/L	[36]
			10–50 µg/L	
As/Rice	2014	Spain, Portugal	0.16 mg/kg	[37]
	2017	Australia, Bangladesh, Korea, India	0.456–1.095 mg/kg	[38]
	2017–2019	India	15–231 μg/kg	[33]
	2018	UK	t-As: 0.01–0.37 mg/kg	[39]
	2018	USA, Canada, KSA, India, Yemen, Iran	t-As: 93–989 µg/kg	[40]
	2020	USA	White rice: 65–202 µg/kg dw Brown rice: 139–403 µg/kg dw Other grains: 1.9–26 µg/kg dw	[41]
	2020	Italy, India, Thailand	White rice: 58–183 µg/kg dw	[41]
As/Fish and Seafood	2006–2010	Norway	Fish: t-As: 0.3–110 mg/kg ww	[42]
	2012	Iran	Fish: 0.168–0.479 µg/g	[43]
	2016	Italy	Fish: 4.89–105.33 ng/g ww	[44]
			Mussels: 15.09–389.62 ng/g ww	
	2017	Greece	Fish: t-As: 11.8–62.6 mg/kg dw	[45]
	2019	Turkey	Fish: 0.24–50.34 mg/kg ww	[46]
	2022	Poland	Fish: 23.3–59,290.1 µg/kg	[47]
As/vegetables	2016	India	Potato: 5.6–176 μg/kg	[33]
As/fruits	2016	Iran	Fruits: 1.279–19.50 μg/kg Fruit juices: 1.137–18.36 μg/kg	[48]
As/milk and dairy products	2015–2016	China	0.004 mg/kg	[49]
As/cereal	2017–2019	India	Wheat flower: 3.6–448 μg/kg	[33]
Hg/Fish and Seafood	2016	Brazil	Bivalve molluscs: 124–725 µg/kg ww	[50]
			Crustacean: 83–149 µg/kg ww	
	2016	Italy	Fish: 5.01–284.94 ng/g ww	[44]
			Mussels: 15.25–480.00 ng/g ww	
	2021	Italy	Fish: THg: 0.03–0.64 µg/g ww	[51]
	2021	Djibouti	Fish: 0.02–1.69 mg/kg ww	[52]
	2022	Poland	Fish: 9.04–606.3 µg/kg	[47]
Hg/Rice	2011	China	THg: 1.8–5 ng/g	[53]
	2014–2015	China	THg: 4.74 µg/kg	[54]
	2014	Sapin, Portugal	Undectable	[37]
	2017	China	THg: 4.03 µg/kg	[55]
			THg: 2.33 µg/kg	
	2017	Pakistan	THg: 4.51 ng/g	[56]
Hg/Wine	2017	Poland	<0.036–0.437 µg/L	[57]
	2018	Poland	0.31–0.51 µg/L	[57]
	2019	France	THg: <0.1–0.55 µg/L	[54]
Hg/juices and fruit juices	2016	Iran	Fruit juices: 0.351 μg/kg Fruit: 690.54 μg/kg	[48]
Hg/Water	2014	Iran	67.41 μg/kg	[58]
Hg/milk and dairy products	2019	India	Milk: 4.88–7.23 µg/kg Cheese: 4.87–8.68 µg/kg Milk powder: 3.34–5.55 µg/kg	[59]
Pb/Fruit and Fruit juices	2014	USA	Apple juice: 3.5 µg/L	[60]
	2014	China	Apple juice: 3.8 µg/L	[60]
	2015	Turkey	Cherry juice: 0.1–16 µg/L	[61]
			Orange juice: 3.7–10 µg/L	
	2016	Portugal	Peach juice: 0.96–8.51 µg/L	[62]
			Orange juice: <LOQ-2.49 µg/L	
	2016 2022	Iran	Fruit juices: 27.87–66.1 μg/kg Fruits: 470.56–910.14 μg/kg	[48]
	2022	Iran	Orange juice: 99 µg/L	[63]
Pb/Vegetables	2007–2016	China	Leafy vegetables: 0.154 mg/kg fw	[64]
			Root vegetables: 0.068 mg/kg fw	
			Fruit vegetables: 0.052 mg/kg fw	
	2016	Iran	Potato: 0.007–0.064 mg/kg ww Onion: 0.002–0.076 mg/kg ww Tomato: <LOD-0.020 mg/kg ww Lettuce: 0.002–0.070 mg/kg ww Leek: 0.005–0.180 mg/kg ww Carrot: 0.006–0.120 mg/kg ww	[65]
Pb/Wheat grains (Cereal grains)	2015	Pakistan	Wheat grain: 0.05–0.29 mg/kg dw	[66]
	2016	Iran	Wheat samples: 0.044 mg/kg	[67]
	2017–2018	Iran	Wheat: 0.027–0.639 mg/kg ww	[65]
	2019	Lebanon	Bread: 74–260 µg/kg dw	[68]
Pb/Milk and dairy products	2015–2016	China	0.0084 mg/kg	[49]
	2019	India	Milk: 9.96–11.89 µg/kg Cheese: 9.16–10.99 µg/kg Milk powder: 3.99–5.01 µg/kg	[59]
	2018	Poland	Milk: 0.012–0.234 mg/kg	[69]
Pb/Rice	2020	USA	White rice: 0.2–31 µg/kg dw Brown rice: 1.4–34 µg/kg dw Other grains: 1.2–80 µg/kg dw	[41]
Pb/Water	2014	Iran	494.12 μg/kg	[58]
Cd/Fish and Seafood	2015	Norway	Crab hepatopancreas: 5.4–16 mg/kg ww	[70]
			Crab claw meat: 0.003–0.006 mg/kg ww	
	2016	Italy	Fish: 5.00–64.96 ng/g ww	[44]
			Mussels: 7.59–97.50 ng/g ww	
	2016–2018	Djibouti	Shark: 0.48 and 14.5 mg/kg	[71]
Cd/Soybean grains	2016	China	0.05 mg/kg dw	[72]
Cd/Vegetables	2021	Cameroon	Cabbage: 0.15 mg/kg Carrot: 0.16 mg/kg	[73]
	2015	Nigeria	Onion: 2.48–6.0 mg/kg	[74]
			Spinach leaves: 0.11–0.21 mg/kg	
			Spinach stems: 0.26–0.31 mg/kg	
	2016	China	Leafy vegetables: 0.01–1.28 mg/kg dw	[75]
			Rootstalk vegetable: <0.01–0.30 mg/kg dw	
			Legume vegetable <0.01–0.04 mg/kg dw	
	2022	India	Mango: 0.01–0.08 mg/kg dw	[76]
Cd/Rice	2014	Sapin, Portugal	Parboiled rice: 0.005 mg/kg	[37]
	2021	Sri Lanka	undetectable-0.1589 mg/kg	[77]
	2016	China	0.02–3.61 mg/kg dw	[75]
	2020	USA	White rice: 1.7–71 µg/kg dw Brown rice: 7.7–65 µg/kg dw Other grains: 1.2–49 µg/kg dw	[41]
Cd/Water	2015	Nigeria	0.09 mg/kg	[74]
	2015	Malaysia	River water: 3.9 × 10^−4^–34.3 × 10^−4^ mg/L	[78]
			Treated water: 1.2 × 10^−4^–9.9 × 10^−4^ mg/L	
			Tap water: 1.3 × 10^−4^–7.7 × 10^−4^ mg/L	
	2017	India	0.05–0.07 mg/L	[79]
Cd/Fruits and Fruit juices	2016	Iran	Fruit juices: 0.89–3.44 μg/kg Fruit: 1.09–5.56 μg/kg	[48] [48]
	2022	Iran	Orange juice: 9.4 µg/L	[63]
Cd/Milk and dairy products	2015–2016	China	0.0097 mg/kg	[49]
	2019	India	Milk: 4.55–8.16 µg/kg Cheese: 3.16–10.93 µg/kg Milk powder: 7.73–10.2 µg/kg	[59]
Cr/Water	2016	UK	maximum value of 15 μg/L, the 95th percentile: 1 μg/L	[80]
	2015	UK	<0.1 μg/L	[81]
	2018	Canada USA	0.1–2 μg/L	[82]
	2015	Malaysia	River water: 1.2 × 10^−4^–12.2 × 10^−4^ mg/L	[78]
			Treated water: 0.2 × 10^−4^–5.3 × 10^−4^ mg/L	
			Tap water: 1.0 × 10^−4^–9.5 × 10^−4^ mg/L	
	2016	Vietnam	Surface water: 0.32–4.32 mg/L	[83]
			Well water: 0.02–0.82 mg/L	
Cr/Vegetables	2018	Romania	17.8 ± 6.9 mg/kg for total Cr	[84]
	2017/2018	India	Cabbage: 0.02–0.46 mg/kg	[85]
			Carrot: 0.21–0.68 mg/kg	
			Onion: 0.12–0.53 mg/kg	
			Tomato: 0.08–0.70 mg/kg	
	2016	Vietnam	0.02–1.57 mg/kg	[83]
Cr/Fish and Seafood	2016	Vietnam	Tiger shrimp: 1.46 ± 0.28 mg/kg	[83]
			Stuffed snails: 1.69 ± 0.40 mg/kg	
			Catfish: 12.25 ± 0.15 mg/kg	
	2016	Italy	Fish: 5.22–109.22 ng/g ww	[44]
			Mussels: 22.54–180.36 ng/g ww	
	2016	Italy	Crab: LOQ 3.216 mg/kg ww	[86]
Cr/Milk and dairy products	2015	France	Milk: 6.2–12 μg/kg fw	[87]
			Cheese: 4.8–101 μg/kg fw	
			Ultra-fresh dairy products: 1.1–21 μg/kg fw	
Cr/Cereal and cereal products	2015	France	Bread and dried bread products: 20–135 μg/kg fw	[87]
			Breakfast cereals: 35–483 μg/kg fw	
			Pasta: Not detected	
			Rice and wheat products: 10–33 μg/kg fw	
			Sweet and savoury biscuits and bars: 20–236 μg/kg fw	

*: year of sampling. **: dw = dry weight; fw = fresh weight; ww = wet weight; t-As = total arsenic; i-As = inorganic arsenic; THg = total mercury; LOQ = limit of quantification.

**Table 2:** Main criteria for the choice of the analysis method for trace element determination (adapted from [177]).

	FAAS	XRF	GAAS	ICP-AES	ICP-MS
Element number					
Single	x	x			
Few			x		
Many				x	x
Element Concentration					
ppm	x	x		x	x
ppb			x	x	x
ppt					x
Sample number					
Very few	x	x	x		
Few	x	x	x	x	x
Many				x	x
Sample volume					
> 5 mL	x	x	x	x	x
< 1–2 mL		x	x		x

FAAS = flame atomic absorption spectrometry; XRF = X-ray fluorescence spectroscopy; GFAAS = graphite furnace atomic absorption spectrometry; ICP-AES = inductively coupled plasma atomic emission spectrometry; ICP-MS = inductively coupled plasma mass spectrometry. x means that the technique is concerned by this criterion.

**Table 3:** Limit of detections of the main essential and toxic elements using the analytical methods: FAAS, XRF, GFAAS, ICP-AES, and ICP-MS.

Z	Essential Elements	FAAS (mg/L)	XRF (mg/L)	GFAAS (µg/L)	ICP-AES (µg/L)	ICP-MS (ng/L)
12	Mg	0.1	600	0.004	0.04	0.01
20	Ca	1.5	8	0.01	0.1	0.05
25	Mn	1.5	10	0.005	3	0.05
26	Fe	5	10	0.06	15	0.1
29	Cu	2	1	0.014	5	0.03
30	Zn	1	9	0.0075	1	0.1
34	Se	50	8	0.03	2	0.3
42	Mo	45	6	0.03	0.5	0.03
Z	Toxic elements	FAAS (mg/L)	XRF (mg/L)	GFAAS (µg/L)	ICP-AES (µg/L)	ICP-MS (ng/L)
33	As	20	10	0.05	15	0.3
46	Pb	20	4	0.04	15	0.01
48	Cd	0.8	20	0.002	1.5	0.06
80	Hg	300	5	0.6	0.5	1

FAAS = flame atomic absorption spectrometry; XRF = X-ray fluorescence spectroscopy; GFAAS = graphite furnace atomic absorption spectrometry; ICP-AES = inductively coupled plasma atomic emission spectrometry; ICP-MS = inductively coupled plasma mass spectrometry. Theoretical detection limits do not take into account interferences. Values collected from several sources.

## Data Availability

No new data were created.
